# Effects of soybean isoflavone aglycone on osteoporosis in ovariectomized rats

**DOI:** 10.3389/fnut.2023.1122045

**Published:** 2023-06-05

**Authors:** Lu-lu Li, Yang Yang, Chun-min Ma, Xiao-mei Li, Xin Bian, Yu Fu, Li-kun Ren, Ru-meng Wang, Yan-guo Shi, Na Zhang

**Affiliations:** ^1^College of Food Engineering, Harbin University of Commerce, Harbin, China; ^2^College of Food Science, Southwest University, Chongqing, China

**Keywords:** soybean, soybean isoflavone aglycone, osteoporosis, serum bone markers, bone microstructure

## Abstract

Postmenopausal osteoporosis is one of the most common metabolic diseases in old women, and supplementing estrogen through bioactive substances is one of the important ways to improve menopausal syndrome. Some studies have confirmed that soybean isoflavone has estrogenic activity, and the main active component of soybean isoflavones is isoflavone aglycones. However, few studies have investigated the improvement effect of high-purity soy isoflavone aglycones on postmenopausal osteoporosis. Thus, the effect of different doses of high-purity soybeans isoflavone aglycone on the ovariectomized female osteoporosis rat model was evaluated by oral gavage. The rats were divided into seven experimental groups including SHAM, OVX, EE, SIHP, AFDP-L, AFDP-M, and AFDP-H, which was administered for 60 days from 30 days after ovariectomy. We collected blood from the abdominal aorta of rats on the 30th, 60th, and 90th days respectively, analyzed its serum biochemistry, and took out the femur for micro-CT imaging and bone microstructure parameter analysis. Results showed that the intervention effect of AFDP-H group on osteoporosis rats at 60 and 90 days was similar to that of EE group, and superior to the OVX group, SIHP group, AFDP-L group, AFDP-M group. The AFDP-H group inhibited the decrease in serum bone markers, bone density, trabeculae quantity, trabeculae thickness, and bone volume fraction, and increased the trabecular separation caused by ovariectomy, thereby significantly improving bone microstructure. It also prevented continuous weight gain and increased cholesterol levels in female rats. This study provided theoretical to application of soybean isoflavone aglycone in the intervention of osteoporosis. and confirmed that could replace chemical synthetic estrogen drugs.

## 1. Introduction

Climacteric syndrome is caused by the deterioration of ovarian function and the deficiency of estrogen before and after menopause of women. Its major symptoms are hot flashes, sweating, insomnia, anxiety, hypertension, reduced immune function, cardiovascular disease and climacteric osteoporosis (1). Based on data provided by the World Health Organization (WHO), there was nearly 460 million women who reached menopause globally in 1999, and by 2030 the number of menopausal women will rise to 1.2 billion, around 80% of whom will get at least one typical symptom of climacteric syndrome. Postmenopausal osteoporosis (PMOP) is a common disease that threats the health of postmenopausal women seriously. The pathogenesis of POMP is involved in many aspects, mainly including the negative balance of bone remodeling, the inhibition of osteoblast proliferation and the reduction of osteoblast differentiation, which can reduce the formation of collagen in bone, resulting in bone loss and bone microstructure deterioration, and then lead to the reduction of bone strength and the increase of fracture risk (2, 3). The present studies have revealed that estrogen is a major driver of bone reconstruction after menopause. Estrogen replacement therapy (ERT) has been used to effectively inhibit the function of osteoclasts and prevent bone loss and fracture in premenopausal and postmenopausal women, but which could increase the risk of breast cancer, ovarian cancer, and endometrial hyperplasia (4, 5). Meanwhile, the WHO International Agency for cancer research issued ERT can induce receptor-mediated tissue-specific and agent-specific cell proliferation, mitogenesis, and other events, thereby increasing the risk of cancer (6). In recent years, researchers are making great efforts to find phytoestrogens that have estrogen effects and do not produce any side effects. Soybean isoflavones (SI), as phytoestrogens, can not only displays estrogen-like activity but also avoid side effects of chemical synthetic estrogen drugs, which are considered as natural substitutes for estrogen drugs and are widely accepted by women (7).

**Figure 1:**
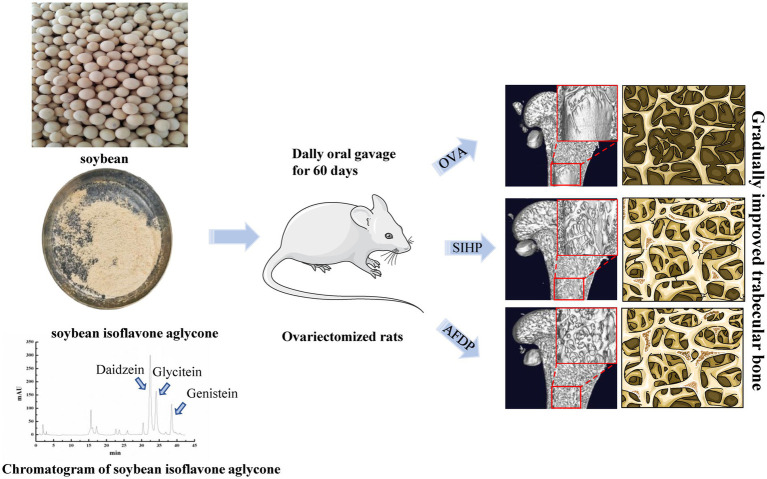
The effects of soybean isoflavones aglycone on ovariectomized rat.

SI is a natural estrogen and a secondary metabolite in the process of soybean formation, which is mainly distributed in the seed coat, hypocotyl and cotyledon of soybean, and its molecular structure is very similar to estrogen in women. Therefore, SI can act like estrogen and exhibit high affinity binding to estrogen receptor (8). Many studies have demonstrated SI can alleviate and improve postmenopausal osteoporosis, reduce the incidence of side effects, and has a biphasic regulatory effect (9, 10). This positive effect is attributed to the fact that the interaction between the bioactive components of SI and estrogen receptors can form estrogen to regulate the expression of osteoblast formation, differentiation, and transcription factors, thereby promoting their proliferation and differentiation to produce necessary nutrients for bone formation and strengthen bone (11, 12). Additionally, SI upregulates osteoprotegerin secreted by osteoblasts, which acts on osteoclast differentiation factors and accelerates osteoclast apoptosis to inhibit bone resorption (13, 14). Many studies have reported that SI were found predominantly in the glycosides form and in low concentrations as aglycones. However, the absorption of free aglycones (daidzein, glycitein, and genistein) in human body is in greater amount than that of conjugated glycosides, and has higher estrogenic activity and antioxidant activity. It has also been demonstrated that the conjugated glycosides cannot be directly absorbed through the small intestine, which should be deglycosylated with the participation of intestinal microorganisms and degraded into corresponding isoflavone aglycone, and finally form relatively stable equol in the body to have biological activity (8, 15). At present, researchers are committed to converting the glycosides of soybean isoflavones into aglycones, but there is a problem of low conversion efficiency. Our research group hydrolyzed the conjugated glycosides of SI through a combination of acid, ultrasound, and enzymatic hydrolysis, and finally obtained the soybean isoflavone aglycone with a purity of 61.84% (Figure 1). However, there are few studies on the effect of soybean isoflavone aglycones with high purity on improving osteoporosis in postmenopausal women.

Here we study the effects of different doses of soybean isoflavone aglycone on body and uterine weight, serum index and bone microstructure parameters for ovariectomized rats as the intervention of osteoporosis. The ability of isoflavone aglycone to inhibit the development of osteoporosis and improve symptoms in ovariectomized rats was evaluated by comparing with the induction effect of estradiol positive drugs and soybean isoflavones health products. Additionally, the effects of different doses of isoflavone aglycone were compared to evaluate the dose–response relationship of aglycone. Our study set out to obtain natural bioactive substances that can replace chemical synthetic estrogen drugs and provide a theoretical basis for the application of soybean isoflavone aglycone in the intervention of osteoporosis.

## 2. Materials and methods

### 2.1. Materials

Soybean isoflavone aglycones (61.84%) freeze-dried powder was provided by our research group (Harbin University of Commerce Harbin, China). Estradiol (2 mg) was purchased from Abbott Healthcare Products (Netherlands). Soy isoflavone health product (purity: 16.37%) was purchased from the Zibo Branch of Huaxia Xietong International Medicine Research Institute (Shandong, China). Normal saline (0.9%) was purchased from Shandong Kelun Pharmaceutical Co., Ltd. (China). Paraformaldehyde (4%) was purchased from ShangHai YuanYe Biotechnology Co., Ltd. (China). Medical alcohol (75%) was purchased from Guilin Lifeng Pharmaceutical Products Co., Ltd. (China). Chloral hydrate was purchased from Tianjin Damao Chemical Reagent Factory (China). Penicillin sodium for injection (4 million units) was purchased from Harbin Pharmaceutical Group Holding Co., Ltd. (China), and the 96 T osteocalcin test kit was purchased from Shanghai Enzyme-linked Biotechnology Co., Ltd. (China).

### 2.2. Experimental animals and treatments

There were 70 eight-week-old specific pathogen-free (SPF) female Sprague–Dawley rats provide by the Changchun Yisi Experimental Animal Center，which were randomly evenly divided into seven groups. And the rats received a bilateral ovariectomy after normal feeding for 7 days according to the method of Saville (16). After 30 days of continuous feeding, each group were infused corresponding drugs, respectively, with 1 mL by oral gavage once daily for 60 days.

The groups were as follows: (1) Sham-operated group (SHAM; negative control, received normal saline orally). (2) Ovariectomized group (OVX; negative control, received normal saline orally). (3) Estradiol group (EE), administered 0.21 mg/kg/day estradiol (dissolved in normal saline). (4) Soybean isoflavone health products group (SIHP), administered 105 mg/kg/day SIHPs (dissolved in normal saline). (5) Low-dose aglycone freeze-dried powder (AFDP-L) group, administered aglycone freeze-dried powder (dissolved in normal saline) at a dose of 24.25 mg/kg/day. (6) Medium-dose aglycone freeze-dried powder (AFDP-M) group, administered aglycone freeze-dried powder (dissolved in normal saline) at a dose of 72.77 mg/kg/day. (7) High-dose aglycone freeze-dried powder (AFDP-H) group, administered aglycone freeze-dried powder (dissolved in normal saline) at a dose of 218.31 mg/kg/day (Table 1).

**Table 1:** Grouping and administration of rats.

Group no.	Gavage samples (1 mL)	Total gavage (mg/kg/d)	Soy isoflavone content (mg/kg/d)	Aglycon content (mg/kg/d)
SHAM	Saline	–	–	–
OVX	Saline	–	–	–
EE	Estradiol	0.21	–	–
SIHP	Soybean isoflavone health products	105.00	17.19	0.30
AFDP-L	Soybean isoflavone aglycone freeze dried powder	24.25	16.01	15.00
AFDP-M	Soybean isoflavone aglycone freeze dried powder	72.77	48.05	45.00
AFDP-H	Soybean isoflavone aglycone freeze dried powder	218.31	144.15	135.00

### 2.3. Measurement of body and uterus weight

The rats were weighed on the 0th (day before castration), 30th, 60th, and 90th days respectively, and the uterus and adipose tissue of rats after death were removed and weighed.

### 2.4. Serum sample tests

We collected blood from the abdominal aorta on the 30th, 60th, and 90th days respectively，which was put into a centrifuge for 4,000 rpm/min for 10 min to separate the serum and then stored in a refrigerator at −20°C. The content of estradiol (E_2_), triglyceride (TG), total cholesterol (TC), bone-derived alkaline phosphatase (ALP), calcium and phosphorus (P) in rat serum were determined by biochemical kits (Nanjing Jiancheng Technology Co., Ltd., China). The content of serum tartrate-resistant acid phosphatase (TRAP) was detected using a DV-5100B UV–vis spectrophotometer (Shanghai Metash Instrument Co., Ltd.). The content of bone Gla protein (BGP) and type I collagen cross-linked N-telopeptide (NXT-I) in rat serum were determined using ELISA kits (Shanghai Enzyme-linked Biotechnology Co., Ltd., China) and the SpectraMax 190 full wavelength light absorption microplate reader (Shenzhen Hicreation Technology Co., Ltd.).

### 2.5. Micro-CT scanning and imaging of the femur and bone microstructure parameters

Bilateral femurs were removed from rats and stored in 4% paraformaldehyde solution at –20°C to obtain the bone microscopic parameters, which were analyzed using a Quantum GX Micro-CT (PerkinElmer Instruments, Inc., United States). The Femur samples were placed in the specimen slots for scanning by the following conditions: voltage 80 kV, current 72 μA, and rotation angle 360°. The 3D structure of each rat femur sample was observed by Micview V2.1.2 3D reconstruction software (17). For comparisons between groups, we selected a region that was located 1–2 mm below the lowest point of the epiphyseal line of the distal femur, and then generated the following index parameters: bone mineral density (BMD), bone volume fraction (BV/TV), trabecular number (Tb.N), trabecular thickness (Tb.Th), and trabecular spacing (Tb.Sp).

### 2.6. Statistical analysis

Statistical analysis was performed by SPSS. All data were expressed as mean ± standard deviation (SD). One-way ANOVA and the LSD multiple range test were used for significance analysis of the differences in measured values among various experimental groups.

## 3. Results and discussion

### 3.1. Organ mass of female rats

#### 3.1.1. The body weight

As shown in Table 2, the body weights of female rats in the SHAM, OVX, EE, SIHP, AFDP-L, AFDP-M, and AFDP-H groups were obtained on 0th, 30th, 60th, and 90th days. There was no significant difference in body weight among the groups at the day 0th, ranging from 182.56 to 191.85 g. Meanwhile, there was no significant difference in body weight of the SHAM group on 30th compared with 0th day, which was due to the influence of castration on appetite and the decrease of food intake, thus resulting in slow growth of body weight. However, compared with the SHAM group, the body weight in the other six groups increased significantly (*p* < 0.05). This was because the estrogen level decreased in ovariectomized rats, and the reduction of estrogen receptor expression reduced signaling through estrogen pathways, which resulted in abnormal blood lipid metabolism, enlargement of fatty cells and increase of fat content, thus promoting fat accumulation and weight gain (18, 19).We could also observe that the weight of rats in the other six groups increased significantly on 60th day than the SHAM group (*p* < 0.05), and the weight gain of the other six groups of rats in a descending order as follows: OVX group, SIHP group, AFDP-L group, AFDP-M group, AFDP-H groups and EE group. On 90th day, the trend of weight gain in these seven groups was consistent with that observed on 60th day. These results indicated that estradiol, soybean isoflavones and aglycones could inhibit abnormal lipid metabolism. These results were consistent with the result of Nogowski et al., who studied the effects of daidzein and genistein on fat synthesis and breakdown in isolated rat adipocytes and found that both aglycones inhibited fat synthesis induced by insulin (20). However, the effect of inhibiting fat synthesis in each group was different on the 90th day, and we could obtain that estradiol and isoflavone aglycones were superior to soy isoflavone in inhibiting weight gain. Furthermore, the inhibition effect was improved with increasing isoflavone aglycones dose. These results further confirmed biological effects of isoflavone in soy are not due to the glycosides form but instead are mainly from their aglycones, including daidzein, genistein and glycitein (chromatogram of soy isoflavone aglycone in Figure 1). Meanwhile, isoflavone aglycones can be absorbed faster and in greater amount than their glycosides (21).

**Table 2:** Body weight, uterine weight and serum index of rats.

Time	Group no.	Weight (g)	Uterine weight (g)	E_2_ (pmol/L)	TG (mmol/L)	TC (mmol/L)
0 d	SHAM	185.74 ± 7.12	–	–	–	–
	OVX	182.56 ± 6.97	–	–	–	–
	EE	189.42 ± 7.85	–	–	–	–
	SIHP	191.85 ± 8.02	–	–	–	–
	AFDP-L	184.73 ± 7.27	–	–	–	–
	AFDP-M	188.06 ± 7.69	–	–	–	–
	AFDP-H	184.94 ± 7.48	–	–	–	–
30 d	SHAM	201.53 ± 7.76	0.58 ± 0.04	237.71 ± 10.61	0.4 ± 0.02	2.88 ± 0.11
	OVX	223.85 ± 8.64^*aa^	0.31 ± 0.02^**^	112.39 ± 5.06^**^	0.39 ± 0.01	1.81 ± 0.05^**^
	EE	227.47 ± 9.12^**aa^	0.32 ± 0.03^**^	115.74 ± 4.49^**^	0.41 ± 0.02	1.91 ± 0.06^**^
	SIHP	229.16 ± 9.35^**aa^	0.34 ± 0.02^**^	122.66 ± 5.61^**^	0.55 ± 0.02^**^	2.24 ± 0.09^**^
	AFDP-L	224.33 ± 8.91^*aa^	0.34 ± 0.02^**^	124.63 ± 6.03^**^	0.49 ± 0.01^**^	3.14 ± 0.10^*^
	AFDP-M	227.61 ± 9.54^**aa^	0.34 ± 0.03^**^	127.38 ± 5.99^**^	0.66 ± 0.02^**^	3.08 ± 0.14^*^
	AFDP-H	226.78 ± 9.28^**aa^	0.37 ± 0.03^**^	142.75 ± 6.34^**^	0.68 ± 0.03^**^	3.19 ± 0.15^**^
60 d	SHAM	214.95 ± 8.74^aab^	0.58 ± 0.05	230.33 ± 9.89	0.45 ± 0.01	2.08 ± 0.09^bb^
	OVX	275.52 ± 12.77^**aabb^	0.10 ± 0.01^**bb^	42.02 ± 1.88^**bb^	0.59 ± 0.02^**bb^	2.73 ± 0.12^**bb^
	EE	239.74 ± 9.89^*▲▲aab^	0.51 ± 0.04^*▲▲bb^	184.14 ± 7.27^**▲▲bb^	0.43 ± 0.02^▲▲^	2.50 ± 0.10^**▲bb^
	SIHP	273.46 ± 11.57^**##aabb^	0.27 ± 0.02^**▲▲##b^	98.33 ± 4.05^**▲▲##bb^	0.48 ± 0.01^▲▲##b^	2.49 ± 0.09^**▲b^
	AFDP-L	269.39 ± 10.89^**#aabb^	0.31 ± 0.02^**▲▲##■b^	111.69 ± 4.96^**▲▲##■b^	0.39 ± 0.02^**▲▲#■■bb^	2.53 ± 0.11^**▲bb^
	AFDP-M	252.94 ± 10.63^**▲#■aabb^	0.33 ± 0.03^**▲▲##■^	120.37 ± 5.53^**▲▲##■■^	0.42 ± 0.01^▲▲■■bb^	2.72 ± 0.13^**bb^
	AFDP-H	242.36 ± 10.18^*▲▲■■aab^	0.41 ± 0.03^**▲▲##■■b^	150.24 ± 6.71^**▲▲##■■^	0.45 ± 0.02^▲▲bb^	2.71 ± 0.12^**bb^
90 d	SHAM	225.57 ± 9.27^aabbc^	0.56 ± 0.04	217.51 ± 10.13	0.43 ± 0.02	2.11 ± 0.10^bb^
	OVX	289.42 ± 11.47^**aabbc^	0.09 ± 0.00^**bb^	31.80 ± 0.97^**bb^	0.71 ± 0.03^**bbcc^	3.45 ± 0.13^**bbcc^
	EE	243.86 ± 10.19^*▲▲aab^	0.54 ± 0.04^▲bb^	201.73 ± 9.74^*▲▲bbc^	0.46 ± 0.02^▲▲^	2.57 ± 0.11^**▲▲bb^
	SIHP	286.03 ± 11.71^**##aabbc^	0.29 ± 0.02^*▲#b^	106.08 ± 4.02^**▲▲##bc^	0.56 ± 0.01^**▲▲##cc^	2.65 ± 0.12^**▲▲bbc^
	AFDP-L	283.15 ± 11.90^**##aabbc^	0.34 ± 0.03^*▲#■c^	123.64 ± 5.63^**▲▲##■^	0.52 ± 0.02^**▲▲##cc^	2.69 ± 0.10^**▲▲bbc^
	AFDP-M	264.79 ± 11.15^**▲##■aabbc^	0.38 ± 0.03^*▲#■bc^	137.52 ± 5.48^**▲▲##■■c^	0.51 ± 0.02^**▲▲#bbcc^	2.71 ± 0.09^**▲▲bb^
	AFDP-H	250.03 ± 10.50^*▲▲■■aabb^	0.49 ± 0.04^*▲#■bbc^	178.91 ± 6.19^**▲▲##■■bbcc^	0.49 ± 0.01^**▲▲■bbc^	2.66 ± 0.07^**▲▲bb^

SHAM, sham operation group; OVX, ovariectomized group; EE, estradiol group; SIHP, soybean isoflavone health products group; AFDP-L, low-dose aglycone freeze-dried powder; AFDP-M, medium-dose aglycone freeze-dried powder; AFDP-H, high-dose aglycone freeze-dried powder. ^a^*p* < 0.05, ^aa^*p* < 0.01, compared of 0 days in each group. ^b^*p* < 0.05, ^bb^*p* < 0.01, Compared of 30 days in each group. ^c^*p* < 0.05, ^cc^*p* < 0.01, compared of 60 days in each group. ^*^p < 0.05, ^**^p < 0.01, compared with SHAM group. ^▲^*p* < 0.05, ^▲▲^*p* < 0.01,compared with OVX group. ^#^*p* < 0.05, ^##^*p* < 0.01, compared with EE group. ^■^*p* < 0.05, ^■■^*p* < 0.01, compared with SIHP group.

#### 3.1.2. The uterus weight

As can be seen from Table 2, there was no significant difference in uterus weight from the 30th to the 90th day in the SHAM group, but in the OVX group, the uterus of rats had obvious atrophy, showing that the uterus weight decreased by 70.97% from the 30th to the 90th day, which caused by estrogen deficiency after ovariectomy. And the study of Kim et al. had also shown the similar results, which showed the weight of uterus after ovariectomy was lower than that of normal female rats, indicating that the uterus gradually atrophied (22). On the 30th day, the uterus weight of the other six groups was significantly lower than that of the SHAM group (*p* < 0.01), but there was no significant difference between the six groups. On the 60th and 90th days, the uterine weight in EE group, SIHP group, AFDP-L groups, AFDP-M groups and AFDP-H group increased compared with that on 30th days, but the EE group and AFDP-H group had a greater increase in uterine weight than the other four groups. Compared with the 30th day, the uterine weight of the AFDP-L group on the 90th day was not significant, but the uterus weight of the AFDP-M group increased (*p* < 0.05), and the uterus weight of the AFDP-H group was significantly increased (*p* < 0.01). These results showed estradiol, soybean isoflavones and aglycones could alleviate the uterine atrophy caused by the significant decrease of estrogen level in ovariectomized rats, and the effects were greatest when given by estradiol and high isoflavones aglycones. Meanwhile, the gavage dose of SIHP was 17.19 g/kg/day, which was similar to the 16.01 g/kg/day found in the AFDP-L group, but the intervention effect of SIHP group was much lower than that in the AFDP-L, AFDP-M, and AFDP-H groups. These results further demonstrated that the biological activity of soy isoflavones was mainly acted from their aglycone, which showed a significant dose–response relationship.

### 3.2. Serum sample index analysis

#### 3.2.1. Hormone levels

As shown in Table 2, the changes in serum estradiol and blood lipid index in rats were determined on 30th, 60th, and 90th days (i.e., 30 and 60 days after drug administration, respectively). From 30th to 90th days, there was no significant difference in serum estradiol levels in the SHAM group, while the estrogen levels in the OVX group decreased significantly by 71.71%. These results further confirmed that ovariectomy had a significant impact on the content of estrogen in rats, and estrogen deficiency led to uterine atrophy, abnormal lipid metabolism, and osteoporosis (23, 24). On the 30th ovariectomy day, the serum estradiol level of ovariectomized rats in all other groups decreased significantly compared with that of the SHAM group (*p* < 0.01), but there was no significant difference between groups. The serum estradiol level of ovariectomized rats in EE group significantly increased from 115.74 to 184.14 pmol/L (*p* < 0.01) on the 60th day, which was 5.24% higher than that of the AFDP-H group. Serum estradiol levels in the AFDP-L and AFDP-M groups were slightly lower than those at 30 days and showed a slight downward trend. The estradiol level in the SIHP group on 60th day was significantly lower than that on 30th day (*p* < 0.01), but significantly higher than that of the OVX group (*p* < 0.01). These results showed that the dose–response relationship of isoflavones aglycone was significant, and the effect of the intervention was limited due to the low isoflavones aglycone content in the mixed soy isoflavone health products. The estradiol levels in each group except the OVX group on 90th day were higher than those on 60th day. Notably, the increase in the EE group was significant (*p* < 0.05) and the increase in the AFDP-H group was highly significant (*p* < 0.01), with the concentration of 201.73 and 178.91 pmol/L, respectively. Meanwhile, the estrogen level on 90th day in the AFDP-M and AFDP-H groups were found to be over the levels observed on 30th day. Although the reduction of estrogen in the SIHP group was inhibited to a certain extent, the effect was significantly lower than that of the AFDP groups (*p* < 0.05). Compared with the OVX group, the estrogen levels in the AFDP-L, AFDP-M, and AFDP-H groups increased by a factor of 2.96, 3.32, and 4.63 times, respectively. This indicated that the hormone level of ovariectomized rats was improved under the intervention of isoflavones aglycone.

#### 3.2.2. Blood lipid index

Meanwhile, we could observe there was no significant change in the level of TG in the SHAM (0.4–0.45 mmol/L) or EE groups (0.41–0.46 mmol/L) on 30, 60, and 90th days, which indicated that the decrease of estrogen level in ovariectomized rats could be alleviated by the intervention of estradiol. The TG levels in the OVX group on 60 and 90th days were significantly higher than those in the SHAM group (*p* < 0.01), which suggested that estrogen deficiency in rats resulted in the upregulation of fatty acid synthase (FAS) gene expression, thereby enhancing the expression of free fatty acids (FFAs) and promoting the synthesis of TG (25). The TG levels in the SIHP, AFDP-L, AFDP-M, and AFDP-H groups were significantly higher at 30 days compared with those in the SHAM group (*p* < 0.01), but decreased significantly from the 30th day to the 60th day (*p* < 0.05), which was decreased by 0.07, 0.10, 0.24, and 0.23 mmol/L in the SIHP, AFDP-L, AFDP-M, and AFDP-H groups, respectively. This indicated that the dosage of isoflavone aglycones is positively correlated with the effect of lowering blood lipid. The TG levels in the SIHP, AFDP-L, AFDP-M, and AFDP-H groups on 90th day returned to the level on the 30th day, which indicated that supplementation of soy isoflavones and aglycone could adjust TG toward normal level, but the isoflavones aglycones showed more positive intervention efficacy.

The TC levels were significantly different between groups (*p* < 0.05) on 30th day, showing that the levels in the OVX and EE groups were significantly lower than those in the SHAM group, while the TC levels in the SIHP, AFDP-L, AFDP-M, and AFDP-H groups were significantly higher than those in the SHAM group. This result might be due to abnormal metabolism caused by feeding and emotional discomfort after surgery and ovariectomy, which made the data on 30th day variable (26). The TC levels in the OVX group had increased by 63.51% from 60th to 90th day, which was significantly higher than those in the SHAM group (*p* < 0.01), and the TC levels on 90th day in the EE, SIHP, AFDP-L, AFDP-M, and AFDP-H groups were significantly lower than those in the OVX group (*p* < 0.01), but remained significantly higher than those in the SHAM group (*p* < 0.01). These results indicated that disordered blood lipid metabolism caused by estrogen deficiency increased the TC levels, and supplementation of estradiol, soy isoflavones, and isoflavones aglycone could improve blood lipid metabolism. However, the TC levels had not returned to normal after administration for 90 days and there was no difference between different groups, indicating that the effect of hormones on reducing TC level was limited.

The explanation of all the above results was that when ovariectomized rats were lack of estrogen, the sensitivity of receptors on the tissue and adipose cell membrane to insulin was reduced, and insulin resistance appeared, which promoted fat mobilization of peripheral tissues, increased the free fatty acids intake by the liver, decreased the activity of lipoprotein lipase, and reduced the oxidation or utilization of the free fatty acids, thus increasing the levels of TG and TC (18, 27). Some researchers have shown estrogen supplementation after ovariectomy regulates the expression of lipid metabolism enzymes in the liver to promote lipid metabolism, thereby increasing the expression of low-density lipoprotein (LDL) receptor and its affinity with LDL, accelerating the uptake and decomposition of LDL by liver cells, and reducing the concentration of TC and LDL in serum. Meanwhile, it also could reduce FAS expression, thus reducing the free fatty acids and the concentrations of serum TG and TC (28). A growing number of menopausal women would choose natural active substances (isoflavone aglycones) to replace estrogen intervention because long-term intake of estrogen could cause thrombosis, cancer and other side effects (2).

### 3.3. Analysis of serum bone markers in rats

As shown in Table 3, the serum calcium (Ca) and phosphorus (P) levels in each group on 30th day had significantly decreased than the SHAM group (*p* < 0.05), but there was no significant difference between the remaining groups. The serum Ca and P levels in the OVX group decreased and were significantly lower than those of the SHAM group (*p* < 0.05) on 60 and 90th days. Studies have shown that the estrogen level influenced serum Ca and P, and the binding ability of Ca and the level of serum P are weakened in the absence of estrogen, resulting in enhanced osteoclast bone resorption, accelerated bone loss, and decreased bone mass (29, 30). Compared with the OVX group, after 60 days of intervention with EE_,_ SIHP and AFDP, the serum Ca and P levels increased significantly (*p* < 0.05), and the intervention effect of AFDP was superior to the SIHP group. These results showed isoflavone aglycone provides the same function as estrogen, which could alleviate the decrease of serum Ca and P levels in ovariectomized rats.

**Table 3:** Serum bone marker index.

Time	Group no.	Ca (mmol/L)	P (mmol/L)	ALP (U/L)	TRAP (U/L)	BGP (ng/mL)	NTX-I (ng/mL)
30 d	SHAM	2.45 ± 0.12	3.89 ± 0.18	92.47 ± 4.43	4.25 ± 0.17	2.07 ± 0.09	8.72 ± 0.38
	OVX	2.26 ± 0.09^*^	3.55 ± 0.16^*^	72.27 ± 3.51^**^	5.83 ± 0.22^**^	3.15 ± 0.10^**^	12.86 ± 0.59^**^
	EE	2.27 ± 0.11^*^	3.25 ± 0.11^*^	72.93 ± 3.39^**^	5.86 ± 0.27^**^	3.06 ± 0.13^**^	13.03 ± 0.64^**^
	SIHP	2.27 ± 0.10^*^	3.17 ± 0.12^*^	73.19 ± 3.46^**^	5.91 ± 0.19^**^	2.96 ± 0.06^**^	13.35 ± 0.56^**^
	AFDP-L	2.25 ± 0.10^*^	3.54 ± 0.11^*^	73.47 ± 3.53^**^	5.94 ± 0.11^**^	3.22 ± 0.12^**^	13.42 ± 0.47^**^
	AFDP-M	2.24 ± 0.08^*^	3.71 ± 0.17^*^	73.66 ± 3.55^**^	5.62 ± 0.28^**^	3.01 ± 0.13^**^	13.63 ± 0.51^**^
	AFDP-H	2.33 ± 0.07^*^	3.46 ± 0.13^*^	74.78 ± 3.63^**^	5.79 ± 0.21^**^	3.18 ± 0.14^**^	13.57 ± 0.60^**^
60 d	SHAM	2.34 ± 0.10^b^	3.65 ± 0.14^b^	91.85 ± 4.39	4.33 ± 0.18	2.13 ± 0.08	9.10 ± 0.44
	OVX	2.16 ± 0.07^*b^	2.45 ± 0.09^**bb^	58.63 ± 2.47^**bb^	6.47 ± 0.29^**b^	3.62 ± 0.15^**bb^	15.09 ± 0.65^**bb^
	EE	2.30 ± 0.09^▲^	3.15 ± 0.13^*▲▲b^	83.91 ± 4.04^*▲▲bb^	4.69 ± 0.22^*▲▲bb^	2.49 ± 0.09^**▲▲bb^	11.56 ± 0.53^**▲▲b^
	SIHP	2.24 ± 0.11^*▲#^	2.83 ± 0.11^**▲▲#b^	68.39 ± 3.22^**▲#b^	5.85 ± 0.23^**▲▲##^	3.27 ± 0.11^**▲▲##b^	14.63 ± 0.71^**▲▲##b^
	AFDP-L	2.25 ± 0.06^*▲#^	2.83 ± 0.12^**▲▲#b^	72.05 ± 3.29^**▲▲#■^	5.43 ± 0.27^**▲▲##■b^	3.03 ± 0.13^**▲▲##■b^	13.21 ± 0.57^**▲▲##■^
	AFDP-M	2.29 ± 0.09^▲■^	2.87 ± 0.10^**▲▲#bb^	72.83 ± 3.53^**▲▲#■^	5.29 ± 0.23^**▲▲#■b^	2.95 ± 0.11^**▲▲##■■^	12.27 ± 0.42^**▲▲#■■b^
	AFDP-H	2.31 ± 0.11^▲■^	3.04 ± 0.14^*▲▲#■b^	79.32 ± 3.84^**▲▲#■b^	4.82 ± 0.20^*▲▲■bb^	2.66 ± 0.07^**▲▲#■■bb^	12.08 ± 0.54^**▲▲#■■b^
90 d	SHAM	2.33 ± 0.10^b^	3.35 ± 0.14^bc^	90.88 ± 4.31	4.39 ± 0.17	2.09 ± 0.09	9.17 ± 0.43
	OVX	2.09 ± 0.08^*bc^	2.31 ± 0.08^**bbc^	51.74 ± 2.09^**bbcc^	6.84 ± 0.31^**bbc^	3.97 ± 0.17^**bbcc^	16.58 ± 0.77^**bbc^
	EE	2.28 ± 0.09^▲^	3.12 ± 0.09^*▲▲b^	86.76 ± 4.21^*▲▲bb^	4.73 ± 0.20^*▲▲bb^	2.63 ± 0.08^**▲▲b^	10.26 ± 0.46^*▲▲bbc^
	SIHP	2.20 ± 0.06^*▲#bc^	2.65 ± 0.10^**▲▲#bc^	70.75 ± 3.13^**▲▲#^	6.22 ± 0.27^**▲##bc^	3.61 ± 0.13^**▲▲##bbcc^	15.33 ± 0.68^**▲##bbc^
	AFDP-L	2.21 ± 0.11^*▲#^	2.74 ± 0.07^**▲▲#■bbc^	73.61 ± 3.49^**▲▲#■^	5.79 ± 0.19^**▲▲##■bc^	3.34 ± 0.07^**▲▲##■c^	13.07 ± 0.53^**▲▲##■■^
	AFDP-M	2.23 ± 0.08^*▲#c^	2.79 ± 0.11^**▲▲#■bb^	75.11 ± 3.56^**▲▲#■^	5.58 ± 0.24^**▲▲##■b^	3.16 ± 0.14^**▲▲##■■c^	12.45 ± 0.41^**▲▲##■■b^
	AFDP-H	2.26 ± 0.10^*▲■b^	2.95 ± 0.12^**▲▲#■b^	82.44 ± 4.17^*▲▲#■bbc^	4.95 ± 0.21^*▲▲■■bb^	2.87 ± 0.10^**▲▲#■■bc^	10.93 ± 0.35^**▲▲#■■bbc^

SHAM, sham operation group; OVX, ovariectomized group; EE, estradiol group; SIHP, soybean isoflavone health products group; AFDP-L, low-dose aglycone freeze-dried powder; AFDP-M, medium-dose aglycone freeze-dried powder; AFDP-H, high-dose aglycone freeze-dried powder. ^b^*p* < 0.05, ^bb^*p* < 0.01, compared of 30 days in each group. ^c^*p* < 0.05, ^cc^*p* < 0.01, compared of 60 days in each group. ^*^p < 0.05, ^**^p < 0.01, compared with SHAM group. ^▲^*p* < 0.05, ^▲▲^ *p* < 0.01,compared with OVX group. ^#^*p* < 0.05, ^##^*p* < 0.01, compared with EE group. ^■^*p* < 0.05, ^■■^*p* < 0.01, compared with SIHP group.

Compared with the SHAM group, the bone alkaline phosphatase (ALP, a typical marker of osteoblasts) levels in all other groups decreased significantly on 30th day (*p* < 0.01), but there was no significant difference between the remaining groups. The ALP levels in the OVX group decreased continuously on 60th day, and compared with the SHAM group, the ALP levels in the OVX group on 90th day had decreased by 43.07%. This was due to the decrease of estrogen levels after ovariectomy, which inhibited the expression of estrogen receptors in osteoblasts, attenuated the activity of osteoblasts, reduced the rate of osteoblast proliferation and differentiation, and finally led to a subsequent decrease in the level of bone ALP (9, 22). After intervention with EE_,_ SIHP and SHAM, the ALP levels increased significantly (*p* < 0.05) compared with those in the OVX group on 60 and 90th days. This indicated that estrogen supplementation could enhance the hydrolysis of phospholipids by ALP during osteogenesis, and the released phosphate and calcium could be deposited on the collagen skeleton to achieve bone formation. Meanwhile, it also promoted the hydrolysis of pyrophosphate by ALP, relieving its inhibition on the formation of bone salt, which was conducive to osteogenesis (31).

The tartrate resistant acid phosphatase (TRAP) levels in the OVX group increased significantly than the SHAM group (*p* < 0.01) on 30th day, but there was no significant difference among the ovariectomized groups. The TRAP levels in OVX group were significantly higher than that in SHAM group on the 60 and 90th day (*p* < 0.01). The research indicated the TRAP levels were involved in the degradation of solid calcium phosphate substrate in bone matrix when bone resorption occurred under estrogen deficiency, which further enhanced the proliferation and differentiation of osteoclasts (31). *In vitro* experiments conducted by Kirstein et al. have revealed that osteoclasts induced the release of abundant TRAP during bone resorption (32). After intervention with EE_,_ SIHP and SHAM, the TRAP levels decreased significantly than the OVX group on 60 and 90th day (*p* < 0.05), which suggested that isoflavone aglycone could reduce the level of TRAP, thus inhibiting the proliferation of osteoclasts and slowing down the development of osteoporosis (9). The TRAP levels in the AFDP-L, AFDP-M, and AFDP-H groups decreased significantly than those in the SIHP group on 90th day (*p* < 0.05), which was due to the higher isoflavone aglycone doses in the AFDP groups than the SIHP group.

The osteocalcin (BGP) levels in all other groups presented a highly significant upward trend (*p* < 0.01) on 30th day besides the SHAM group, and there was no significant difference between ovariectomized groups. The BGP levels in the OVX group were significantly higher than those of the SHAM group on 60 and 90th days (*p* < 0.01), which was due to the fact the osteoporosis model established after ovariectomy in female rats had the characteristics of high bone turnover rate, and the BGP levels was significantly increased. After intervention with EE_,_ SIHP and AFDP, the BGP levels significantly decreased on 60 and 90th day than those in the OVX group (*p* < 0.01), which suggested that intervention could reduce the BGP levels, attenuate bone resorption, and promote bone formation.

The collagen I cross-linked amino terminal peptide (NTX-I) levels in the other groups had increased significantly (*p* < 0.05) on 30th day than the SHAM group, and there was no significant difference between the ovariectomized groups. The NTX-I levels in the OVX group had increased continuously on 60th day, and the NTX-I level in the OVX group had increased by 80.81% compared with that in the SHAM group on 90th day. This was due to the decreased levels of estrogen and the increased number of osteoclasts after ovariectomy, which increased the blood levels of NTX-I (29). After intervention with EE_,_ SIHP and AFDH, the serum NTX-I levels decreased significantly (*p* < 0.05) than those in the OVX group. Rosen et al. reported similar results, which indicated that the bone mineral density of women with the lowest NTX-I concentration increased significantly than those with the highest NTX-I concentration among 239 postmenopausal women who received ERT or calcium therapy (33).

In the analysis of serum bone markers, we found that the AFDP group could improve the abnormal level of serum bone markers and reduce the symptoms of osteoporosis compared with SIHP group, and isoflavone aglycone can be used as a substitute of estrogen drugs to intervene osteoporosis in ovariectomized rats.

### 3.4. Microstructure observation and parameter analysis of rat femur by micro-CT

**Figure 2:**
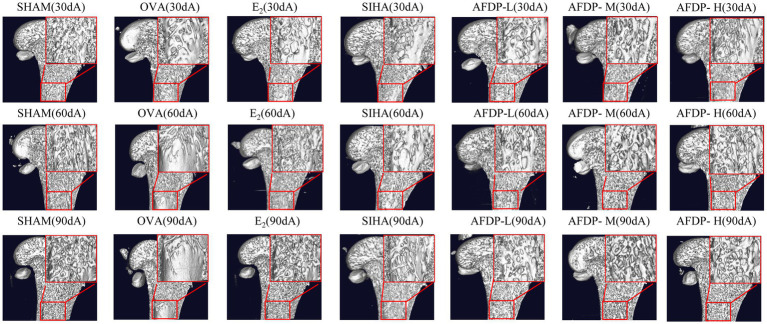
Application of micro-CT in trabecular imaging of distal femur. “30 d, 60 d, 90 d” represent the 30 days, 60 days, 90 days. “A ~ G” represent the sham operation group, ovariectomized group, estradiol group, soybean isoflavone health products group, low-dose soybean isoflavone aglycone freeze-dried powder, medium-dose soybean isoflavone aglycone freeze-dried powder, high-dose soybean isoflavone aglycone freeze-dried powder.

There were several imaging techniques to study animal bone tissue morphology, including Micro-CT, magnetic resonance microscopy, high-frequency ultrasound imaging and micro-positron emission tomography (34, 35). Recently, the Micro-CT has been widely used in non-invasive and anatomical imaging of small animal tissues or organs due to its advantages of high spatial resolution and high sensitivity to bones (36). The femurs of rats in the seven groups were scanned using Micro-CT, and changes in the microstructure were evaluated on 30th, 60th and 90th day. As shown in Table 4 and Figure 2, there was no significant change in the five indexes of bone microstructure in the SHAM group on 30, 60, and 90th days, which could be observed using Micro-CT that the trabeculae were evenly distributed and arranged in a tight network. Instead, the BMD, BV/TV, Tb.N, and Tb.Th in the OVX, EE, SIHP, AFDP-L, AFDP-M, and AFDP-H groups on 30th day decreased significantly (*p* < 0.05, *p* < 0.01), while the Tb.Sp was increased significantly (*p* < 0.01), which showed that the rats in the OVX, EE, SIHP, AFDP-L, AFDP-M, and AFDP-H groups had bone loss 30 days after castration. Over time, the BMD decreased by 18.00%, the BV/TV and Tb, N decreased by 91.93 and 86.77% respectively, and the Tb.Sp increased by 79.89% in OVX group on 90th day. Figure 2 (90 dB) showed that bone loss mainly occurred in trabeculae, and the connection of bone trabeculae was interrupted and nearly hollow in the OVX group. This was due to the reduction of estrogen caused by bilateral ovariectomy in rats, which inhibited the expression of estrogen receptors on osteoblasts and osteoclasts, made it difficult to regulate gene transcription, reduced the direct impact on osteoblasts and osteoclasts and led to the hyperfunction of osteoclasts and bone resorption exceeding bone formation, thus promoting the development of osteoporosis (37, 38).

**Table 4:** Serum bone marker index.

Time	Group no.	BMD (g/cm^3^)	BV/TV (%)	Tb.N (mm^−1^)	Tb.Th (mm)	Tb.Sp (mm)
30 d	SHAM	13.4559 ± 0.6118	33.1137 ± 1.6254	4.5709 ± 0.2135	0.1318 ± 0.0059	0.1775 ± 0.0086
	OVX	11.9903 ± 0.4523^*^	17.4983 ± 0.8172^**^	2.1796 ± 0.0912^**^	0.0935 ± 0.0036^**^	0.2609 ± 0.0121^**^
	EE	12.0189 ± 0.4832^*^	17.6477 ± 0.8217^**^	2.1811 ± 0.0929^**^	0.0943 ± 0.0039^**^	0.2591 ± 0.0117^**^
	SIHP	12.1125 ± 0.5771^*^	17.7365 ± 0.8344^**^	2.1769 ± 0.0908^**^	0.0962 ± 0.0043^**^	0.2577 ± 0.0119^**^
	AFDP-L	12.1252 ± 0.5429^*^	17.5633 ± 0.7985^**^	2.1932 ± 0.0933^**^	0.0971 ± 0.0038^**^	0.2572 ± 0.0120^**^
	AFDP-M	12.1386 ± 0.5837^*^	17.3732 ± 0.7877^**^	2.1943 ± 0.1017^**^	0.0988 ± 0.0045^**^	0.2569 ± 0.0116^**^
	AFDP-H	12.1524 ± 0.5692^*^	17.8763 ± 0.8069^**^	2.1966 ± 0.1023^**^	0.1023 ± 0.0047^**^	0.2571 ± 0.0125^**^
60 d	SHAM	13.3740 ± 0.5289	33.0915 ± 1.5928	4.5433 ± 0.2074	0.1313 ± 0.0055	0.1765 ± 0.0081
	OVX	11.1562 ± 0.4882^**b^	4.4056 ± 0.2027^**bb^	0.8124 ± 0.0394^**bb^	0.0645 ± 0.0029^**bb^	0.3118 ± 0.0148^**bb^
	EE	12.8391 ± 0.5317^*▲▲b^	27.4537 ± 0.1330^**▲▲bb^	3.5720 ± 0.1685^**▲▲bb^	0.1188 ± 0.0054^*▲▲b^	0.2091 ± 0.0093^**▲▲bb^
	SIHP	11.8232 ± 0.4566^**▲#b^	13.5372 ± 0.6731^**▲▲##bb^	1.9058 ± 0.0856^**▲▲##b^	0.0845 ± 0.0041^**▲##b^	0.2711 ± 0.0128^**▲▲##b^
	AFDP-L	11.9745 ± 0.4824^*▲#^	15.7107 ± 0.7385^**▲▲##■b^	2.1663 ± 0.1031^**▲▲##■^	0.0892 ± 0.0035^**▲##b^	0.2618 ± 0.0124^**▲▲##^
	AFDP-M	12.0424 ± 0.5152^*▲#■^	17.1218 ± 0.8156^**▲▲##■■^	2.1922 ± 0.1129^**▲▲##■^	0.0923 ± 0.0037^**▲▲##■b^	0.2577 ± 0.0122^**▲▲##■^
	AFDP-H	12.4763 ± 0.5337^*▲■b^	21.9552 ± 1.0715^**▲▲##■■bb^	2.9137 ± 0.1386^**▲▲##■■bb^	0.1053 ± 0.0049^**▲▲#■^	0.2312 ± 0.0109^**▲▲#■■bb^
90 d	SHAM	13.2371 ± 0.4424	32.871 ± 1.5653	4.4993 ± 0.1986	0.1305 ± 0.0046	0.1753 ± 0.0073
	OVX	11.0335 ± 0.3872^**b^	2.6711 ± 0.1292^**bbc^	0.6047 ± 0.0296^**bbc^	0.0661 ± 0.0026^**bb^	0.3193 ± 0.0151^**bb^
	EE	13.0747 ± 0.4187^▲▲bc^	30.3084 ± 1.4596^*▲▲bbc^	3.9098 ± 0.1890^**▲▲bbc^	0.1220 ± 0.0059^*▲▲bbcc^	0.1947 ± 0.0092^▲▲bbc^
	SIHP	11.8996 ± 0.4059^*▲#b^	14.8023 ± 0.7301^**▲▲##bc^	2.0573 ± 0.1019^**▲▲##c^	0.0887 ± 0.0039^**▲##b^	0.2664 ± 0.0127^**▲▲##b^
	AFDP-L	12.1265 ± 0.4204^*▲#^	17.6408 ± 0.8739^**▲▲##■c^	2.1971 ± 0.1146^**▲▲##^	0.0945 ± 0.0047^**▲▲##^	0.2526 ± 0.0119^**▲▲##^
	AFDP-M	12.2882 ± 0.3933^*▲#■c^	19.8953 ± 0.9166^**▲▲##■■bc^	2.6671 ± 0.1251^**▲▲##■■bc^	0.0997 ± 0.0044^**▲▲##■c^	0.2427 ± 0.0115^**▲▲##■b^
	AFDP-H	12.7759 ± 0.4737^*▲▲■bc^	26.9497 ± 1.2374^**▲▲##■■bbcc^	3.4985 ± 0.1704^**▲▲##■■bbccc^	0.1170 ± 0.0057^*▲▲■■bbcc^	0.2129 ± 0.0101^**▲▲■■bbc^

SHAM, sham operation group; OVX, ovariectomized group; EE, estradiol group; SIHP, soybean isoflavone health products group; AFDP-L, low-dose aglycone freeze-dried powder; AFDP-M, medium-dose aglycone freeze-dried powder; AFDP-H, high-dose aglycone freeze-dried powder. ^b^*p* < 0.05, ^bb^*p* < 0.01, compared of 30 days in each group. ^c^*p* < 0.05, ^cc^*p* < 0.01, compared of 60 days in each group. ^*^p < 0.05, ^**^p < 0.01, compared with SHAM group. ^▲^*p* < 0.05,^▲▲^*p* < 0.01,compared with OVX group. ^#^*p* < 0.05, ^##^*p* < 0.01, compared with EE group. ^■^*p* < 0.05, ^■■^*p* < 0.01, compared with SIHP group.

As shown in Table 4 and Figure 2 (60 dC, 90 dC), the BMD, BV/TV, Tb.N, and Tb.Th in the EE group on 60 and 90th days increased significantly (*p* < 0.01) than the OVX group, while Tb.Sp decreased to 0.2091 and 0.1947. The bone tissue structure of the distal femur was repaired, and the tissue morphology was uniform, which indicated that the trabecular microstructure of rats was improved after ingestion of estradiol. Previous studies have reported that estradiol had a good effect in improving female menopausal osteoporosis, but long-term use of it would lead to side effects of cardiovascular disease, increasing the risk of breast hyperplasia and uterine fibroids (39). Therefore, the use of estrogen drugs for intervention in female climacteric syndrome, including osteoporosis, has been decreased considerably in recent years.

Compared with the OVX group, the BMD, BV/TV, Tb.N, and Tb.Th in the SIHP group on 60 and 90th days increased significantly (*p* < 0.05, *p* < 0.01), while the Tb.Sp decreased significantly (*p* < 0.01). However, TB.Sp in the SHAM group was significantly higher on 90th days than on 30th days (*p* < 0.01), while the other four parameters were significantly lower. This indicated that the SIHP group had the effect of estrogen, which could inhibit the decrease of bone trabecular microstructure parameters caused by estrogen deficiency, but none of the femoral microscopic parameters returned to the levels observed in the SHAM group and the degree of improvement of osteoporosis in the SIHP group was far lower than that in the EE group. This was because the low concentration of isoflavone aglycones has less biological activity. In the AFDP-L group, the BMD, BV/TV, Tb.N, and Tb.Th increased significantly (*p* < 0.05, *p* < 0.01) on 60 and 90th days, while the Tb.Sp decreased significantly (*p* < 0.01) than those of the OVX group. However, there was no significant difference in the BMD, Tb.N and TB.Sp on 60 and 90th day between the AFDP-L and SIHP group. At 90 days after administration, these parameter values were almost same as those observed at 30 days after oophorectomy, but their effects on osteoporosis were not as good as the AFDP-M and AFDP-H groups. In the AFDP-M group, the BMD, BV/TV, Tb.N, Tb.Th, and Tb.Sp were improved than the AFDP-L group on 60 and 90th days, and the data on 60th day were highly similar to those observed 30 days after ovariectomy. Generally, the effects in the AFDP-M group were greater than those in the AFDP-L group. In the AFDP-H group, the BMD, BV/TV, Tb.N, Tb.Th, and Tb.Sp were all improved than the AFDP-M group on 60 and 90th days, and were closer to the levels observed in the SHAM group. Meanwhile, there was no significant difference in BMD, Tb.Th, and Tb.Sp than the EE group.

Compared with other groups, the bone tissue structure of EE (90dC), AFDP-M (90dF) and AFDP-H (90 dG) groups improved significantly within 90 days, and was the closest to the level observed in the SHAM group, which was consistent with the parameters observed in the micro-CT imaging (Figure 2). Ahn and Park studied soy isoflavone interventions (total isoflavone content: 21.1%) at low (10 mg/kg·d) and high (50 mg/kg·d) doses for 8 weeks and found that long-term supplementation with soy isoflavones had positive effects on BV/TV, Tb.N, and Tb.Th of rats, but had no effect on the level of the CTX level (9). Conversely, supplementing isoflavone aglycone (45.00 mg/kg·d) for 8 weeks can significantly reduce CTX level and improve the tibia bone quality by increasing BV/TV, Tb.N, and Tb.Th. These results indicated that high-dose isoflavone aglycone and estradiol have similar efficacy, the biological activity of isoflavone aglycone is higher than that of soybean isoflavones, and the isoflavone aglycone alleviates osteoporosis in a dose-dependent manner.

## 4. Conclusion

The effects and mechanisms of isoflavone aglycone on osteoporosis in ovariectomized rats were studied by animal experiments. It was found that isoflavone aglycone could reduce the levels of TC, TG, ALP, BGP, NTX-I, Tb.Sp, and increase the levels of Ca, P, BAP, BMD, Tb.Th, BV/TV, Tb.N. The results showed that isoflavone aglycones (AFDP-M and AFDP-L) could partly improve the phenomenon of excessive weight gain in obese rats, and improve serum lipid indicators and bone markers at a certain extent. These results fully indicated that isoflavone aglycone could be used to intervene and treat osteoporosis in ovariectomized rats, inhibited obesity, and prevented hyperlipidemia caused by abnormal lipid metabolism. Therefore, the bioactive isoflavone aglycones can replace estrogen in the treatment of postmenopausal osteoporosis, which provided an interesting and novel method for the clinical treatment of osteoporosis caused by estrogen deficiency.

## Data availability statement

The original contributions presented in the study are included in the article/supplementary material, further inquiries can be directed to the corresponding author.

## Ethics statement

The animal study was reviewed and approved by the Experimental Animal Ethics Sub-Committee, School of Food Engineering, Harbin University of Commerce.

## Author contributions

NZ: supervision and project administration. LL, YY, and CM: methodology, validation, and writing-original draft and editing. XL and XB: formal analysis. YF, LR, and RW: data curation and writing-review. YS: methodology and validation. All authors contributed to the article and approved the submitted version.

## Funding

This work was supported by the National Natural Science Foundation (Nos. 31871747 and 32072258) and the National Key Research and Development Program of China (No. 2016YFD0400402).

## Conflict of interest

The authors declare that the research was conducted in the absence of any commercial or financial relationships that could be construed as a potential conflict of interest.

## Publisher’s note

All claims expressed in this article are solely those of the authors and do not necessarily represent those of their affiliated organizations, or those of the publisher, the editors and the reviewers. Any product that may be evaluated in this article, or claim that may be made by its manufacturer, is not guaranteed or endorsed by the publisher.
